# Gut Fecal Microbiota Transplant in a Mouse Model of Orthotopic Rectal Cancer

**DOI:** 10.3389/fonc.2020.568012

**Published:** 2020-10-28

**Authors:** Yen-Cheng Chen, Zhi-Feng Miao, Kwan-Ling Yip, Yi-An Cheng, Chung-Jung Liu, Ling-Hui Li, Chung-Yen Lin, Jiunn-Wei Wang, Deng-Chyang Wu, Tian-Lu Cheng, Jaw-Yuan Wang

**Affiliations:** ^1^ Division of Colorectal Surgery, Department of Surgery, Kaohsiung Medical University Hospital, Kaohsiung Medical University, Kaohsiung, Taiwan; ^2^ Department of Biomedical Science and Environmental Biology, Kaohsiung Medical University, Kaohsiung, Taiwan; ^3^ Drug Development and Value Creation Research Center, Kaohsiung Medical University, Kaohsiung, Taiwan; ^4^ Division of Gastroenterology, Department of Internal Medicine, Kaohsiung Medical University Hospital, Kaohsiung, Taiwan; ^5^ Institute of Biomedical Sciences, Academia Sinica, Taipei, Taiwan; ^6^ Institute of Information Science, Academia Sinica, Taipei, Taiwan; ^7^ Department of Medicine, College of Medicine, Kaohsiung Medical University, Kaohsiung, Taiwan; ^8^ College of Medicine, Graduate Institute of Medicine, Kaohsiung Medical University, Kaohsiung, Taiwan; ^9^ Department of Surgery, Faculty of Medicine, College of Medicine, Kaohsiung Medical University Hospital, Kaohsiung Medical University, Kaohsiung, Taiwan; ^10^ Graduate Institute of Clinical Medicine, College of Medicine, Kaohsiung Medical University, Kaohsiung, Taiwan; ^11^ Center for Cancer Research, Kaohsiung Medical University, Kaohsiung, Taiwan; ^12^ Center for Liquid Biopsy and Cohort Research, Kaohsiung Medical University, Kaohsiung, Taiwan

**Keywords:** orthotopic rectal cancer model, fecal microbiota transplant, colorectal cancer, *Acinetobacter lwoffii*, *Bifidobacterium longum*

## Abstract

The gut microbiota is reported to play an important role in carcinogenesis and the treatment of CRC. SW480 and SW620 colon cancer cells integrated with infrared fluorescent proteins were injected into the rectal submucosa of nude mice. In the subsequent 30 days, we observed tumor growth weekly using an *in vivo* imaging system. The bacterial solution was infused anally into the mice to perform bacterial transplant. Phosphate-buffered saline, *Acinetobacter lwoffii*, and *Bifidobacterium longum* solutions were infused individually. The 16S ribosomal DNA (rDNA) and polymerase chain reaction of murine feces were investigated to confirm the colonization of target bacteria. In the SW620 orthotopic xenograft rectal cancer model, 4 of 5 mice developed rectal cancer by 30 days after submucosal injection. In the SW480 orthotopic xenograft rectal cancer model, 2 of 6 mice developed rectal cancer by 30 days after submucosal injection. For the 16S rDNA analysis, the mice receiving the bacterial solution infusion demonstrated positive findings for *A. lwoffii* and *B. longum*. With the successful establishment of a mouse model of orthotopic rectal cancer and transplant of target bacteria, we can further explore the relationship between gut microbiota and CRC. The role of fecal microbiota transplant in the treatment and alleviation of adverse events of chemotherapy in CRC could be clarified in subsequent studies.

## Introduction

Colorectal cancer (CRC) now is the fourth most commonly diagnosed malignancy and the third leading cause of cancer-related death worldwide (1, 2). Globally, the number of CRC cases has been increasing gradually. Asia has contributed to half of the incidence and mortality. Moreover, the mortality rate remains high in developed countries such as Japan, China, South Korea, and Taiwan (2). Hence, investigations have focused on the mechanism and treatment of CRC. The human gut microbiota was discovered to be associated with the tumorigenesis of CRC. The pathogenesis pathway includes toxins, inflammation, oxidative stress, and microbially derived metabolism (3–5), and some carcinogenic pathway–associated and pathogen-associated microbiota have been found. For example, Enterococcus faecalis, Escherichia coli, Bacteroides fragilis, and Helicobacter pylori were found to have some interaction with the gut immune system and be capable of inducing CRC (3, 4, 6, 7). However, some bacteria involved in normal human metabolism have a therapeutic effect on CRC (3–5, 7). The interaction between gut microbiota and CRC is still being explored.

Considering the therapeutic effect of the gut microbiota, some researchers have focused on the development of fecal microbiota transplantation (FMT), the concept of which is thought to be first introduced approximately 1,700 years ago in Chinese medicine (6, 8). In 1983, FMT was successfully applied in pseudomembranous colitis with the use of commensal bacteria to replace the harmful Clostridium difficile (8, 9). The efficacy of FMT is well established in the treatment of pseudomembranous colitis, including administration methods and donor screening criteria (8–10). Because the gut microbiota is supposedly associated with human CRC carcinogenesis, an increasing number of studies have focused on the anticancer effects of FMT.

Many animal models have been established to clarify the effects of different gut microbiota. Colon cancer animal models are essential to testing and verifying hypotheses. Ectopic tumor models with subcutaneous injection of CRC cells are the simplest and most frequently used models; however, ectopic models cannot mimic the real microenvironment (11, 12). Instead, with portal and systemic blood supply, the environment of the orthotopic CRC model is more similar to real pathophysiology and therefore enables the observation of cancer lymph nodes or distant metastases (11, 13–16). Submucosal injection of tumor cells has been proven to be effective for establishing a CRC mouse model. Compared with open laparotomy, submucosal injection is a less challenging technique (13–17). Through the use of noninvasive imaging techniques with fluorescence and bioluminescence, tumor growth can be recorded continuously without necessitating that animals be sacrificed for histological analysis (11, 12).

Orthotopic CRC model has some advantages such as use of human cancer cells, metastatic potential and with lymphovascular invasion. Compared with other available models, orthotopic CRC can better investigate the tumor microenvironment (18). With this study, we present our preliminary data of target bacteria transplant in an orthotopic rectal cancer mouse model. Two-part experiments were performed. The first part was the establishment of orthotopic rectal cancer in nude mice. The second part was transanal infusion of two target bacterial solution, demonstrating the application of FMT. By using these animal models, we may further investigate the pathogenic or protective effects of specific bacterial species on CRC tumors in mice.

## Material and Methods

### Cell Line and Culture Conditions

SW480 and SW620 cells comprise two human colon adenocarcinoma cell lines, which we purchased from the American Type Culture Collection (ATCC; Manassas, VA, USA). These cells are tumorigenic in nude mice. We used RPMI 1640 culture medium and 10% fetal calf serum and added 1% penicillin-streptomycin and L-glutamine (Invitrogen, Karlsruhe, Germany) as supplementation. The cell strain was cultured in a glass flask, with a cell coverage rate of 85–90%. We stored the cell strain in an ultralow-temperature refrigerator (constant temperature 37°C; containing 5% CO2 humidified gas).

### Animals and Animal Care

Male and female nude mice were purchased from Taiwan National Laboratory Animal Center (Taipei City, Taiwan). The mice were aged 4–6 weeks. After acclimatization for 1 week, the mice were subjected to the experiment. The animal laboratory conditions met the specific pathogen free (SPF) criteria. The mice were housed in plastic cages. The room temperature was maintained at approximately 20°C, and the humidity was approximately 45%. We fed the mice an *in vivo* imaging diet (BioLASCO Taiwan Co., Ltd., Taipei City, Taiwan), which can decrease chlorophyll fluorescence and thus can detect tumors clearly through imaging. Each mouse had ad libitum access to food and water, and the day/night cycle was 12/12 h. The animal experiment protocol was approved by the animal use committee of our university.

### Preparation of Cell Suspension for Injection

After the integration of infrared fluorescent protein (iFP) DNA sequences into pLKO-AS2 plasmid (National RNAi Core Facility, Academia Sinica, Taipei, Taiwan), the iFP-expressing plasmid was transfected into human colon cancer SW480 and SW620 cells, which were harvested from near-confluent cultures through brief (3 min) exposure to 0.5% trypsin and 0.02% ethylenediaminetetraacetic acid (EDTA) (Invitrogen). Trypsinization was stopped with Dulbecco’s modified Eagle medium (DMEM) containing 10% fetal bovine serum (FBS), and the cells were concentrated through centrifugation and resuspended in DMEM containing 10% FBS. Trypan blue staining was used to assess cell viability, and only cell suspensions consisting of single cells with >90% viability were used for the injections. The concentration of the SW480 and SW620 suspensions was 8×10^6^ cells/50 μl.

### Transanal Submucosal Injection of Colon Cancer Cells

Nude mice were anesthetized through chloroform inhalation. We fixed the mice on a 45× magnification Leica Zoom 2000 stereomicroscope (Leica Microsystems, Buffalo Grove, IL, USA) and then gently dilated their anus with blunt-tipped forceps. SW480 and SW620 cells were suspended in DMEM with 10% FBS, and we used a 30-gage syringe for injection. After we identified the rectum, 50-μl suspensions containing 8×10^6^ SW480 and SW620 cells were smoothly injected on the distal posterior rectal submucosa, respectively. Injection was performed at an angle of 60 degrees approximately 1–2 mm above the anal canal. Injection below the anal canal would lead to an unfavorable anal tumor model. We also avoided transmural injection to prevent the development of a pelvic cavity tumor model. After injection, the mice were observed for 30 min and then monitored three times weekly to measure the tumor size. Thirty days after injection, the mice were sacrificed so that we could examine the rectal tumors. For the control group, the procedure was the same, but we used phosphate-buffered saline (PBS) as the submucosal injection.

### Liposome Solution and Imaging System

Liposomes labeled with 1,1-dioctadecyl-3,3,3,3-tetramethylindodicarbocyanine (DiD) fluorescent dye (FormuMax Scientific, Inc., Sunnyvale, CA, USA) were used to assist with transanal infusion of the bacterial solution. We diluted liposomes 1:500 in PBS, and then we infused the solution through the anus of the mice. By detecting fluorescence, we evaluated the status of solution retention in the murine gut. Ten minutes after solution infusion, we used an *in vivo* imaging system (IVIS) (PerkinElmer, Inc., Waltham, MA, USA) to detect the liposome solution.

### Bacterial Solution and Transanal Infusion

The target bacteria, *Bifidobacterium longum* and *Acinetobacter lwoffii*, were selected with reference to our unpublished data. We analyzed patients with stage IV colorectal cancer who had received chemotherapy with irinotecan plus target agents. The stool samples from these patients with severe diarrhea and no diarrhea were collected. A 16S rRNA analysis revealed that *B. longum* may have a preventative effect on diarrhea and that *A. lwoffii* may worsen diarrhea. In addition, a literature review revealed that *B. longum* is a well-recognized probiotic. In mouse experiments, *B. longum* has been demonstrated to prevent epithelial barrier impairment and relieve inflammation, thus demonstrating its ability to reduce murine colitis (19–21). In mice with irinotecan-induced small intestine mucositis, *B. longum* can also relieve symptoms (22). The antitumor effects of *B. longum* have also been reported (23). However, some literature has indicated a relationship between *A. lwoffii* and gastritis (24, 25). We considered that *A. lwoffii* and *B. longum* may have opposite roles in colorectal cancer treatment. Thus, we chose these two bacteria as target bacteria for further studies.

These bacteria were purchased from ATCC. As a bacterial solution, these bacteria were suspended in PBS at a concentration of 1.5×10^8^ cfu/ml. Nude mice were anesthetized through chloroform inhalation. We fixed the mice on the operating table in supine position with the head down at a 45-degree angle. After identifying the anus of the mice, we used feeding needle with a blunt tip to collect the bacterial solution. The needle was gently inserted approximately 5 cm deep into the anus, and then transanal infusion was performed with 40 μl of solution on every mouse.

A total of six nude mice were used. PBS was infused into two mice as the control group. *A. Iwoffii* and *B. longum* solution was infused in two mice separately. All mice received one infusion of solution daily on three successive days. After transanal infusion, we collected stool from all six mice from day 4 to day 18 for further analysis.

### 16S Ribosomal Deoxyribonucleic Acid Analysis

#### Extraction of Genomic DNA

Total genomic DNA from mouse fecal samples was extracted using the ZymoBIOMICS DNA Miniprep Kit (Zymo Research, Irvine, CA, USA) according to the manufacturer’s instructions.

#### Amplicon Generation

Primers used for *A. lwoffii* were as follows: forward, 5′-TGGCTCAGATTGAACGCTGGCGGC-3′; reverse, 5′-TACCTTGTTACGACTTCACCCCA-3′. Primers used for *B. longum* were as follows: forward, 5′-TTCCAGTTGATCGCATGGTC-3′; reverse, 5′-GGAAGCCGTATCTCTACGA-3′. All polymerase chain reactions (PCRs) were conducted in 30-μl reactions with 15 μl of GoTaq^®^ Green Master Mix (Promega, Madison, WI, USA), with 0.2 μM forward and reverse primers and approximately 10 ng of genomic DNA. Thermal cycling for amplification of *A. lwoffii* DNA began with the initial denaturation step at 95°C for 5 min, followed by 35 cycles of denaturation at 95°C for 45 s, annealing at 67°C for 45 s, and elongation at 72°C for 60 s, and finally at 72°C for 7 min. Thermal cycling for the amplification of *B. longum* DNA began with the initial denaturation step at 94°C for 5 min, followed by 40 cycles of denaturation at 94°C for 20 s, annealing at 55°C for 20 s, and elongation at 72°C for 50 s, and finally at 94°C for 15s.

#### Agarose Gel Electrophoresis for PCR Products

Agarose gel electrophoresis was performed on 0.7% agarose gel (SeaKem^®^ LE Agarose; Lonza, Morristown, NJ, USA) with 0.5× Tris-acetate-EDTA as an electrophoresis buffer. Prior to cool-down of the boiled agarose, EtB”Out” Nucleic Acid Staining Solution (5 μl; YB Biotech, Taipei City, Taiwan) was added to liquid agarose (100 ml) for visualization of the separated DNA bands under ultraviolet light after electrophoresis. The DNA sample was loaded into the wells with bromophenol blue dye. The power condition was set as 130 V and 400 mA, and electrophoresis proceeded for 20 min. The DNA bands were finally photographed under ultraviolet light.

## Results

### Development of Orthotopic Rectal Cancer in Nude Mice

#### SW620

**Figure 1:**
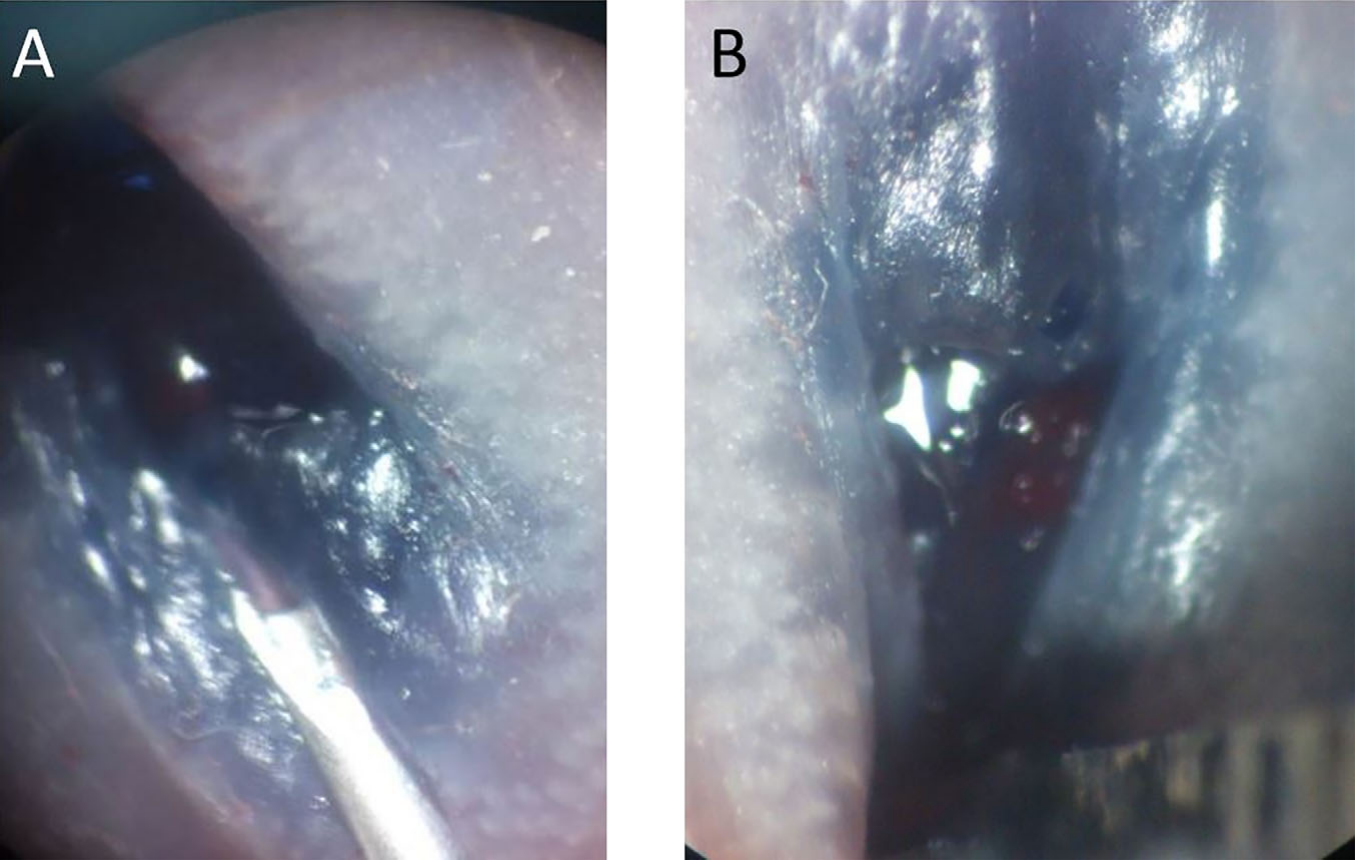
**(A)** Blue dye was mixed with cell suspension to facilitate localization of the submucosal injection. **(B)** After injection, mild enlargement of the submucosal area was observed in the murine rectum.

**Figure 2:**
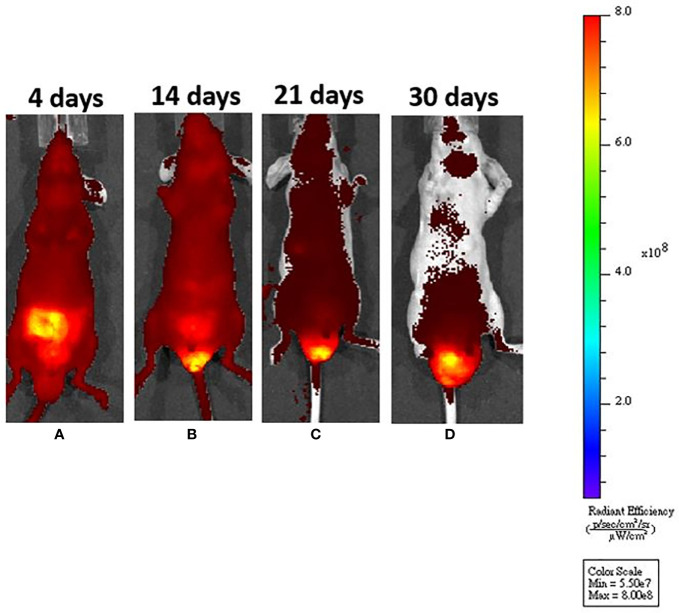
After submucosal injection, we could identify tumor growth by *in vivo* system imaging. **(A)** On day 4, fluorescence interference from a normal diet was noted, but there was no fluorescence around the anus. **(B)** On day 14, the fluorescence interference decreased after mice were fed an *in vivo* imaging diet. An enhanced fluorescence signal could be seen around the anus. **(C, D)** The fluorescence signal was increased, demonstrating tumor progression around the murine anus.

**Figure 3:**
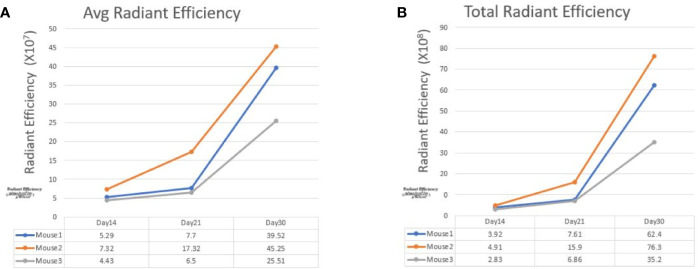
The line graphs display the fluorescence radiant efficiency from 3 SW620 mice. In both average and total radiant efficiency, the fluorescence gradually increased from day 14 to day 30. This result indicated tumor growth after transanal injection of cancer cells. The details of fluorescence radiant efficiency were described in the table below.

**Figure 4:**
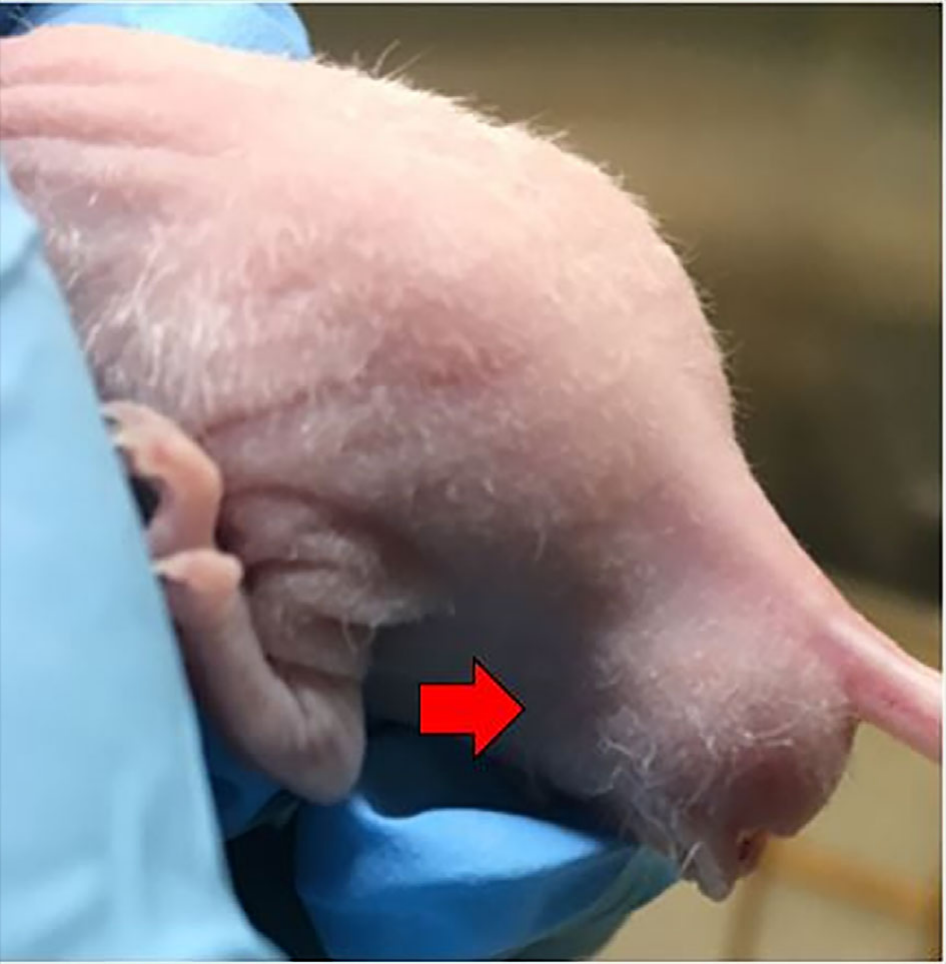
Successful establishment of rectal cancer could be identified by the enlarged, firm mass around the murine anus (red arrow).

**Figure 5:**
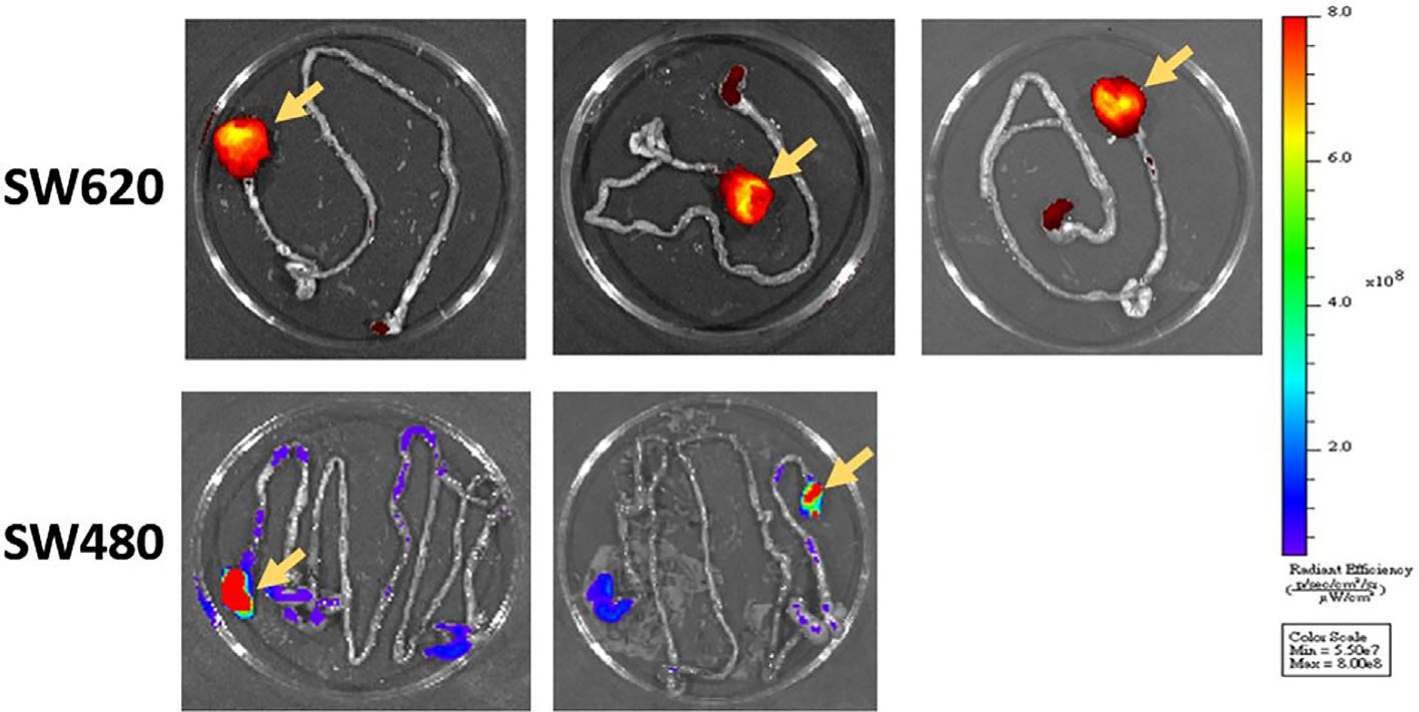
After the mice were killed, we confirmed tumor formation through gross examination and IVIS imaging. The primary rectal tumor was identified as that with the higher fluorescence intensity (yellow arrows).

**Figure 6:**
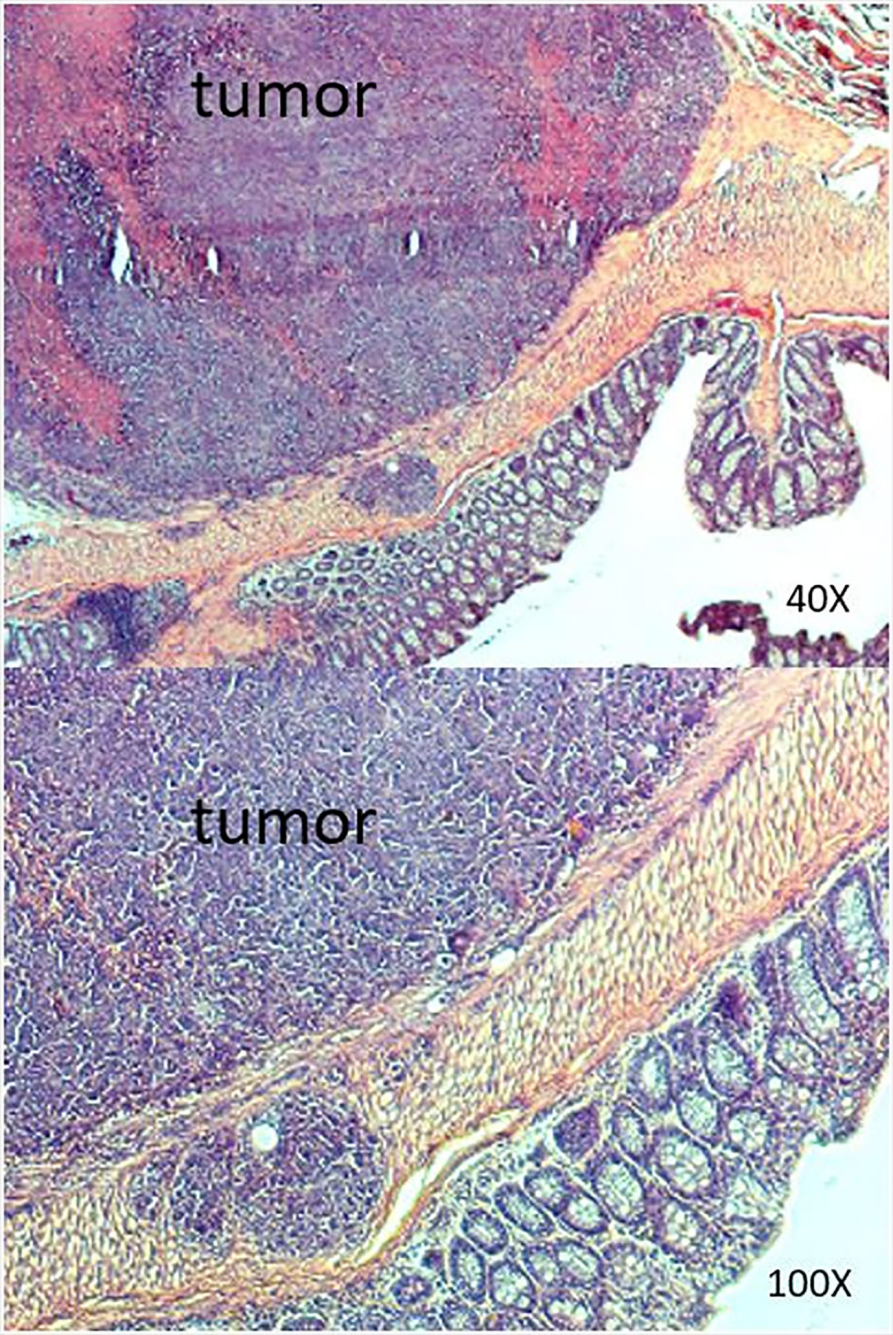
Results of histopathologic examination of the murine intestine from the SW620 group. Adenocarcinoma was detected.

We successfully performed submucosal injection into five nude mice. SW620 cells were implanted on the distal rectum (**Figures 1A, B**). After submucosal injection of SW620 cells, we used an IVIS to observe tumor growth weekly. We identified tumor growth with IVIS. From days 14 to 30, the images demonstrated tumor progression around the anus (**Figure 2**). The line graphs displayed in **Figures 3A, B** also demonstrated gradually enhanced fluorescence. Mice were sacrificed on the 31st day, and rectal cancer could be observed with an enlarged mice anus (**Figure 4**). By contrast, the control group displayed a normal appearance. We confirmed tumor growth through gross examination using IVIS (**Figure 5**) and histopathological examination (**Figure 6**). A total of five mice received submucosal injections, and four mice developed orthotopic rectal cancer. The overall orthotopic rectal cancer–development success rate among the mice was thus 80%.

#### SW480

Six nude mice were administered a submucosal injection. We confirmed tumor growth by gross examination using IVIS (**Figure 5**) and histopathological examination. The other experimental details were the same as those for SW620 cells. However, only two mice developed orthotopic rectal cancer after submucosal injection. The success rate was only 33%.

#### Control Group

One nude mouse was administered a submucosal injection with PBS as a control. From days 4 to 30, IVIS imaging revealed no signs of tumor development (**Supplementary Figure 1**). Histopathological examination also confirmed this negative finding (**Supplementary Figure 2**).

### Pretest of Transanal Infusion of Target Bacteria by Using Liposomes

**Figure 7:**
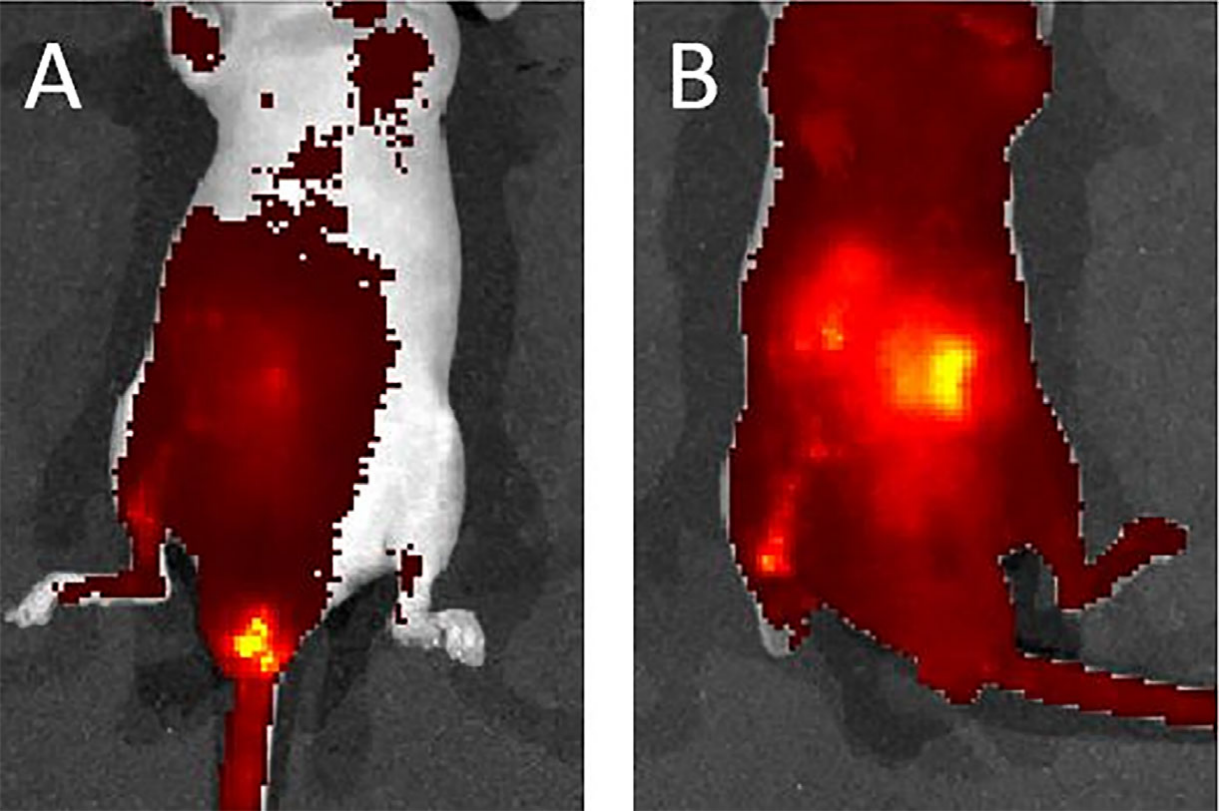
**(A)** One minute after liposome infusion, the IVIS detected the solution around the murine anus. **(B)** Ten minutes later, the IVIS revealed a fluorescent signal across the whole abdomen. This image suggests a good distribution of the solution.

We used a liposome solution as a pretest of bacterial transplantation. In the mice that received oral liposome feeding, after 24 h, IVIS imaging still captured a clear image. This result suggested that the liposome structure was stable in the murine intestinal tract (**Supplementary Figure 3**). After transanal infusion of 40 μl of liposome solution labeled with DiD fluorescent dye. We used an IVIS to detect the distribution of the solution. One minute after infusion, the IVIS revealed solution localization around the anus. However, 10 min later, imaging indicated fluorescent signaling in the whole abdomen, which suggested good distribution of the solution (**Figures 7A, B**). On the basis of this satisfactory result with the liposome solution, we infused the bacterial solution of *A. lwoffii* or *B. longum* the same way. After transanal infusion for three successive days, the feces from six mice were collected every day for 14 days for 16S ribosomal deoxyribonucleic acid (rDNA) analysis.

### Bacterial DNA Analysis

**Supplementary Table 1** summarizes the primer information for agarose gel electrophoresis of *A. lwoffii* and *B. longum*. We analyzed the bacterial solution as a positive control (**Supplementary figure 4**). *A. lwoffii* DNA amplified with *A. lwoffii* primer (PA) exhibited a high signal of approximately 1,500 bp (lane 1), but no signal was detected with *B. longum* primer (PB) (lane 2). Instead, *B. longum* DNA amplified with primer PB revealed high signal over 831 bp (lane 3) and no signal with primer PA (lane 4). This finding indicated the unique specificity of the two primer pairs for the detection of *A. lwoffii* and *B. longum*. This picture demonstrated a standard positive finding.

**Figure 8:**
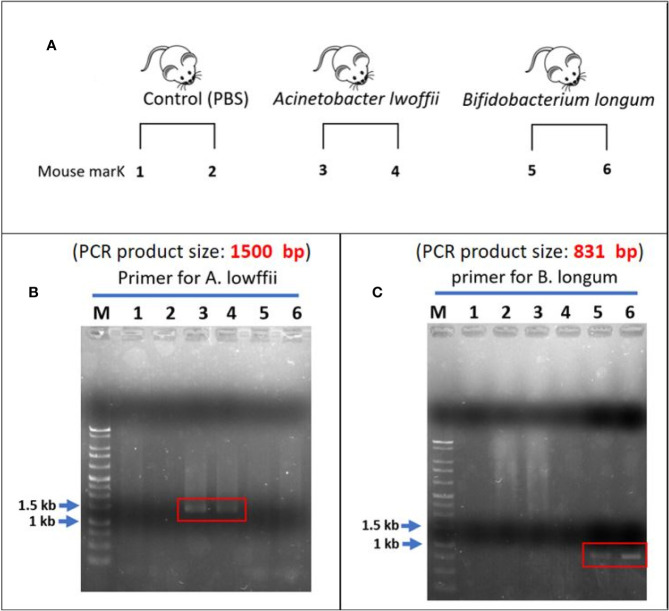
**(A)** Experimental design. **(B)** Lanes 1 and 2 are the control group. After the infusion of phosphate-buffered saline, there was no enhanced signal across the whole lanes. In the *A.* *lwoffii* group, the fecal analysis revealed an enhanced signal of approximately 1,500 bp in lanes 3 and 4. There was no enhanced signal in the lanes 5 and 6. This finding indicated that both mice that received *A.* *lwoffii* infusion exhibited good colonization of the target bacteria. **(C)** Negative findings can also be noted in lanes 1 and 2. An enhanced signal of approximately 831 bp appeared in lanes 5 and 6. Instead, there was no signal on lanes 3 and 4. This finding indicated good colonization of *B.* *longum* in the two mice that received transanal infusion of the *B.* *longum* solution. The negative finding in the control group showed that *A.* *lwoffii* and *B.* *longum* were acquired rather than natural gut flora.

The experimental design is presented in **Figure 8A**. In fecal analysis, the two mice that received *A. lwoffii* solution infusion exhibited enhanced signaling around 1,500 bp in lanes 3 and 4 but no signal in lanes 5 or 6 (**Figure 8B**). Similarly, the two mice that received *B. longum* solution infusion exhibited enhanced signaling around 831 bp in lanes 5 and 6 but no signaling in lanes 3 or 4 (**Figure 8C**). Both lanes 1 and 2 presented negative findings at 1,500 bp and 831 bp, which indicated that *A. lwoffii* and *B. longum* were acquired rather than natural gut flora. These findings indicated good colonization of the target bacteria after transanal bacterial infusion.

## Discussion

This study presents an orthotopic xenograft rectal cancer animal model and a method of applying transplanted target bacteria in an SPF environment. The following are characteristics of this animal model: (1) successful orthotopic rectal cancer murine model developed through submucosal injection of human colon cancer cells, (2) noninvasive observation of tumor growth through the IVIS, and (3) successful bacterial transplant through transanal infusion. During investigation of the mechanism of specific bacteria, germ-free conditions and the use of mice are useful for limiting interference from other bacterial species. For example, *Campylobacter jejuni* was proved to induce colitis and subsequent CRC tumorigenesis in a germ-free animal model (26–28). However, maintenance of germ-free or gnotobiotic conditions can be expensive (29, 30). By contrast, a conventional mouse model with cocktail antibiotic pretreatment is also feasible means of investigating specific bacterial species (13, 15, 17). In the present study, all animal experiments were performed in a standard SPF environment. Without antibiotic pretreatment, we still established an orthotopic rectal cancer model with bacterial transplantation. However, this model cannot completely clarify the pathogenetic or therapeutic effects of specific gut flora. Thus, we will include antibiotic pretreatment in future experiments.

There are many methods of inducing CRC in mice, including chemical approaches, the transgenic method, and implantation (11, 12). Dextran sulfate sodium causes mouse colitis and can predispose transgenic animals to colon carcinogenesis, such as in mice with adenomatous polyposis coli (APCMin/+) (11, 12, 27). However, these models have limitations because the tumors do not have human colon cancer cells. To explore the pathogenic pathway of specific bacteria, implantation of human colon cells in immunosuppressive nude mice would be an ideal method (11, 12). Previous studies have described an open surgical method of implanting human colon cells into mice (12, 31–33); nevertheless, this method involves challenging techniques and operative complications (12, 31). Transanal submucosal injection is nonoperative and is less invasive than the open surgical method (12, 13, 16, 17).

In the present study, one mouse in SW620 group failed to develop orthotopic rectal cancer after submucosal injection. The reason might be cell suspension leakage. Under a stereomicroscope, the visual field is narrow, and there is no parameter for measuring the depth of needle injection. Combined with an amateur skill level of the person performing the injection, insufficient injection depth can cause leakage of cell suspension. However, too great of an injection depth can cause intraperitoneal injection, trauma, or animal death. Hence, submucosal injection is a procedure that requires practice. The dentate line and murine rectum must be identified, and then the mucosa of the murine distal rectum must be clearly located; therefore, adequate magnification equipment is necessary to perform submucosal injection. Some researchers have also suggested the use of a microscope rather than the naked eye to achieve a higher success rate (16, 17). In the experimental protocol we followed, the injection point was 2 mm above the dentate line, and the needle was inserted at an angle of 60 degrees and depth of approximately 3 mm. These details of our protocol can help researchers perform adequate submucosal injection. Some literature has recommended low-volume injection of an approximately 10 μl cell suspension. High-volume injection of approximately 50 μl may cause tissue pressure in the submucosal area, which can lead to cell suspension leakage through the tract when the needle is withdrawn. This may lead to unfavorable outcomes (16). Although a 50-μl injection volume seemed feasible for our purposes, we may use a low-volume injection of approximately 10 μl in future experiments to achieve a better outcome.

Even with the same technique, the success rate in the SW480 group was below 50%. One possible reason is differences in cancer cell characteristics. Although derived from a single patient, SW620 cells are more tumorigenic and metastatic than SW480 cells. As a metastasis-derived cell line, SW620 cells exhibit less susceptibility to apoptosis (34). These features indicate that SW620 can be a better candidate cell to establish an orthotopic rectal cancer model, which could be more feasible in a subsequent FMT animal model.

Surgical resection remains the major therapy for CRC, and multimodal therapy has been applied in a recent treatment programs as adjuvant or neoadjuvant therapy. Nevertheless, metastatic CRC is still a clinical challenge because approximately 20% of patients are initially diagnosed with metastatic CRC (35–37). Reports have indicated that approximately 40% of patients with CRC who receive curative therapy have tumor recurrence and distant metastasis (35, 36). Therefore, many researchers have attempted to improve the treatment outcomes, and human gut microbiota may be a potential solution (3–8). The human gut microbiota is a complex community, containing over 100 trillion microbial cells (3, 38, 39), and it was found to be involved in many aspects of normal human physiology. Gut microbiota has a positive effect on pathogen protection, nutrient supplementation, immunomodulation, and host metabolism adjustment (3, 4). The diversity and balance of the intestinal microbiota, referred to as homeostasis, is essential to maintaining these beneficial interactions to improve human health (3–5). Instead, the disturbance of microbial diversity, called dysbiosis, can lead to harmful effects, including inflammatory bowel disease, hepatic disease, diabetes mellitus, nephropathy, microbiota–gut–brain axis disease, and cancer (3–6).

Increasing evidence has demonstrated that the alteration of gut bacteria is related to the success of disease treatment (3–6). The gut microbiota can affect the outcome of cancer treatments, including radiotherapy (40), chemotherapy (41), and immunotherapy (42–44), as well as their side effects (8). For instance, the gut microbiota can have a synergistic effect with cyclophosphamide (CTX) (8, 42, 43). CTX is used as chemotherapy or immunosuppressive therapy and can break the intestinal barrier and lead to discontinuities and disconnection of intestinal barrier. Specific gram-positive bacterial species subsequently translocate into lymphoid organs and can enhance the response of cytotoxic and helper T cells, thereby improving the anticancer effect of CTX. In contrast, broad-spectrum antibiotics have adverse effects on gut microbiota, thus compromising CTX treatment response (8, 42, 43).

Regarding CRC, the gut microbiota was proved to be associated with tumorigenesis by toxins, inflammation, oxidative stress, and microbially derived metabolism (3–6). Some carcinogenic pathways and pathogens have been found. *E. faecalis*, *E. coli*, *F. nucleatum*, *H. pylori*, and *B. fragilis* were found to have some interaction with the gut immune system and be capable of inducing CRC tumorigenesis (3–8). For example, *F. nucleatum* was reported to have adverse effects on colon cancer treatment and be capable of inducing chemoresistance by modulating autophagy, thereby reducing patient survival duration and promoting colon cancer recurrence (8, 41). *F. nucleatum* was also found to activate β-catenin signaling and induce oncogenic and inflammatory response, thereby promoting CRC cell growth (45).

FMT is a procedure of transplanting the gut microbiota from healthy donors to sick patients. This procedure was first applied to restore microbial diversity in patients with pseudomembranous colitis (6, 8, 9). Recently, the criteria for FMT in patients with *C. difficile* infections have been well established (9, 10, 46), but the application of FMT for the treatment of CRC is still under development. With more evidence derived from research, the effects of microbiota composition and FMT on CRC tumorigenesis and its potential in therapeutic strategies are attractive topics worthy of further investigation (3, 4, 8, 9). Some bacterial species, such as *Lactobacillus* and *Bifidobacterium*, were found to reconstruct gut microbial diversity and improve dysbiosis in patients with CRC. These results indicate the potential anticancer treatment effect of probiotics (6, 8, 47, 48). There are many methods of performing FMT. Through the upper gut, probiotics can be ingested in capsule form or directly in liquid form. Through the midgut, probiotics can be delivered through gastroscopy to the duodenum or by tube feeding, such as through a nasojejunal tube, percutaneous gastrostomy, or jejunostomy. Through the lower gut, an enema is the simplest method. An enema can be performed through the anus or a stoma. Colonoscopy or colonic transendoscopic enteral tubing can also be used to infuse probiotics (8, 9). However, there is no consensus regarding the most effective method for performing FMT.

The human gut microbiota can be transplanted to a mouse to induce carcinogenesis (49, 50). After the gavage of fecal samples from healthy individuals and patients with cancer, those from patients with cancer induced more polyps and mucosal dysplasia (49, 50). In addition, specific pathogens are highly related to tumorigenesis (49, 50). However, the period required for feeding mice can reach 2–3 weeks (49, 50). In our unpublished work, we tried to identify a relationship between target bacteria and irinotecan-induced diarrhea. We established a mouse model with oral feeding of *A. lwoffii* and *B. longum*. However, both groups exhibited severe diarrhea with intestinal mucosa atrophy. Contrary to our expectations, we failed to observe obvious protective effects of *B. longum* against diarrhea. We speculated that possible reasons were too many interfering factors of oral feeding and the unpredictability of the digestion process. Gastric juice, biliary juice, and intestinal fluid may have affected the results. In addition, the condition of bacterial colonization around the rectum may have important effect on rectal cancer cell. Therefore, we chose transanal infusion rather than oral feeding in this bacterial transplant animal model. The subsequent bacterial DNA analysis of feces also revealed positive findings of target bacterial DNA. This result indicated good colonization of the target bacteria. Even with only three applications of transanal infusion, the bacteria were transplanted successfully into the murine gut without decay. This result indicates that transanal infusion may be a more effective method for implanting specific probiotics or pathogens.

In the present study, we used 16S rDNA to confirm the colonization of the two target bacteria. To detect *A. lwoffii* and *B. longum*, PCR analysis of 16S rDNA with species-specific primers is thought to be a quick and sensitive method (51, 52). For bacteria that are difficult to culture, PCR analysis of 16S rDNA is an effective method to prove the presence of specific gut flora. Take *B. longum* as an example, if the bacterial concentration exceeds 10^6^ cells per gram of feces, it can be detected by PCR (51, 52). However, PCR is unable to determine the predominant bacteria in a cluster of gut microbiota (51, 52). Because the pathogenetic or therapeutic effect is thought to be promoted by a cluster of bacteria, next-generation sequencing is necessary to validate these effects in future studies.

An IVIS has been performed to assess tumor growth, cancer metastasis, and treatment effects in animal models. The major advantage of this approach is the ability to observe *in vivo* sequential images of living animals. Noninvasive management is easier to perform and can prevent experimental animals from being sacrificed (11, 53, 54). In the present model, an IVIS was used not only in an orthotopic rectal cancer model but also for the evaluation of bacterial transplant. Liposomes have been widely used in drug testing as a delivery vehicle because they are biocompatible and biodegradable but do not induce immune response (55). In the present study, liposomes marked with DiD fluorescent dye were prepared in a solution. This solution was infused as a pretest of bacterial implantation. Through assessment using an IVIS, we evaluated the solution distribution and modified the infusion technique to confirm solution retention without passage or leakage with the stool.

The animal model had some limitations. First, the number of nude mice was insufficient. Second, although the success rate of the SW620 tumor model reached 80%, only 5 mice were used in the experimental group to obtain the preliminary results of the establishment in the orthotopic animal model. Because of the limited number of mice, the stool volume was insufficient to present the changes of bacterial colonization in sequence. The condition of bacterial retention and decay is unclear. In future studies, with the experience gained from this study and an increased number of mice, we can further improve the success rate and establish more detailed results. Third, our animal model was used in two separate experiments as a prototype. We could not identify any treatment or tumorigenic effect on our target bacteria. The combination of these two models may be useful in future work. Transanal infusion of target bacteria to mice with orthotopic rectal cancer can be our next step for exploring the relationship between the gut microbiota and CRC.

In this study, we present an FMT animal model in two stages: first, orthotopic xenograft rectal cancer in mice; second, bacterial transplant with *A. lwoffii* and *B. longum* through transanal infusion. Because the gut microbiota has been proven to have potential to alter the CRC treatment effects and affect the occurrence of adverse events, our team aims to establish data regarding possible pathogens or probiotics in CRC in Taiwan in the future by using this FMT animal model to further investigate the interaction between rectal cancer and target bacteria. The establishment of animal models is only the beginning of investigation into FMT for the treatment of CRC.

## Conclusion

Submucosal injection is a method of establishing an orthotopic xenograft rectal cancer model. With the assistance of an IVIS, tumor location and growth can be observed noninvasively. Transanal infusion is an effective method for delivering target bacteria and achieving adequate colonization. The combination of these two models is a promising approach to the development of an animal model of FMT for treating CRC for reference in future studies.

## Data Availability Statement

The raw data supporting the conclusions of this article will be made available by the authors, without undue reservation.

## Ethics Statement

The animal study was reviewed and approved by Animal Use Committee of Kaohsiung Medical University.

## Author Contributions

J-YW, T-LC, D-CW, C-YL, and L-HL conceptualized the study. Z-FM, K-LY, Y-AC, C-JL, and J-WW investigated the study. Y-CC, Z-FM, K-LY, Y-AC, and C-JL contributed to methodology. Y-CC, Z-FM, and C-JL wrote the original draft. Y-CC, Z-FM, J-YW, T-LC, and L-HL reviewed and edited the manuscript. J-YW, T-LC, Y-CC, Z-FM, D-CW, C-YL, and L-HL approved the final version of manuscript to be published. All authors contributed to the article and approved the submitted version.

## Funding

This work was supported by grants through funding from the Ministry of Science and Technology (MOST 109-2314-B-037-035, MOST 109-2314-B-037-040, MOST 109-2314-B-037-046-MY3) and the Ministry of Health and Welfare (MOHW107-TDU-B-212-123006, MOHW107-TDU-B-212-114026B, MOHW108-TDU-B-212-133006, MOHW109-TDU-B-212-134026, MOHW109-TDU-B-212-114006) and funded funding by from the health and welfare surcharge of on tobacco products, and the Kaohsiung Medical University Hospital (KMUH108-8R34, KMUH108-8R35, KMUH108-8M33, KMUH108-8M35, KMUH108-8M36, KMUH-DK109003, KMUH-DK109005~3, KMUHS10903) and KMU Center for Cancer Research (KMU-TC108A04) as well as and a KMU Center for Liquid Biopsy and Cohort Research Center Grant (KMU-TC109B05), Kaohsiung Medical University. In addition, this study was supported by the Grant of Taiwan Precision Medicine Initiative, Academia Sinica, Taiwan, R.O.C.

## Conflict of Interest

The authors declare that the research was conducted in the absence of any commercial or financial relationships that could be construed as a potential conflict of interest.

## Supplementary Material

Supplementary Figure 1 This image is the control group of the orthotopic rectal cancer model. PBS solution (50 μl) was injected into the submucosal layer of the rectum through the anus. The IVIS image presented shows no signs of tumor growth; only fluorescence interference was recorded.

Supplementary Figure 2 Histopathologic examination of murine intestines from the control group. Intact intestinal gland and submucosal structure are presented. There were no signs of adenocarcinoma.

Supplementary Figure 3 Twenty-four hours after oral administration of the liposome solution with DiD fluorescent dye, fluorescence could be detected. The result suggested that the liposome structure was stable in the murine intestinal tract.

Supplementary Figure 4 The positive control exhibited a standard image after DNA analysis. The *Acinetobacter Iwoffii* DNA marker appeared at 1,500 bp, and *Bifidobacterium longum* appeared at 831 bp.
