# Supplementary material for: Gut Fecal Microbiota Transplant in a Mouse Model of Orthotopic Rectal Cancer
Source: Front Oncol. 2020 Oct 28;10:568012. doi: 10.3389/fonc.2020.568012 (PMC7658813; doi:10.3389/fonc.2020.568012)
Supplement: Supplementary Figure 1 — This image is the control group of the orthotopic rectal cancer model. PBS solution (50 μl) was injected into the submucosal layer of the rectum through the anus. The IVIS image presented shows no signs of tumor growth; only fluorescence interference was recorded. [file DataSheet_1.docx]

Supplementary Material

**Supplementary Figure 1**
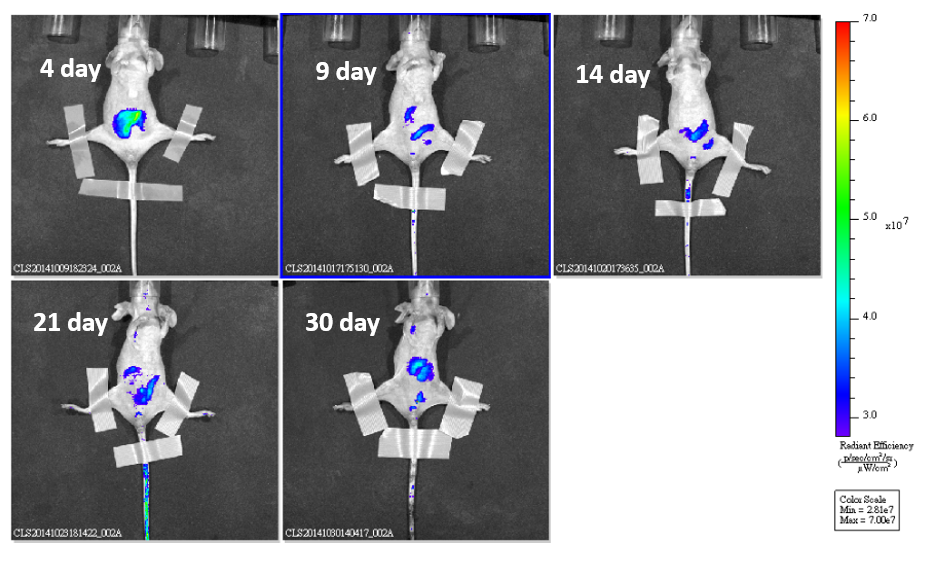


Supplementary Figure 1

This image is the control group of the orthotopic rectal cancer model. PBS solution (50 μL) was injected into the submucosal layer of the rectum through the anus. The IVIS image presented shows no signs of tumor growth; only fluorescence interference was recorded.

**Supplementary Figure 2**
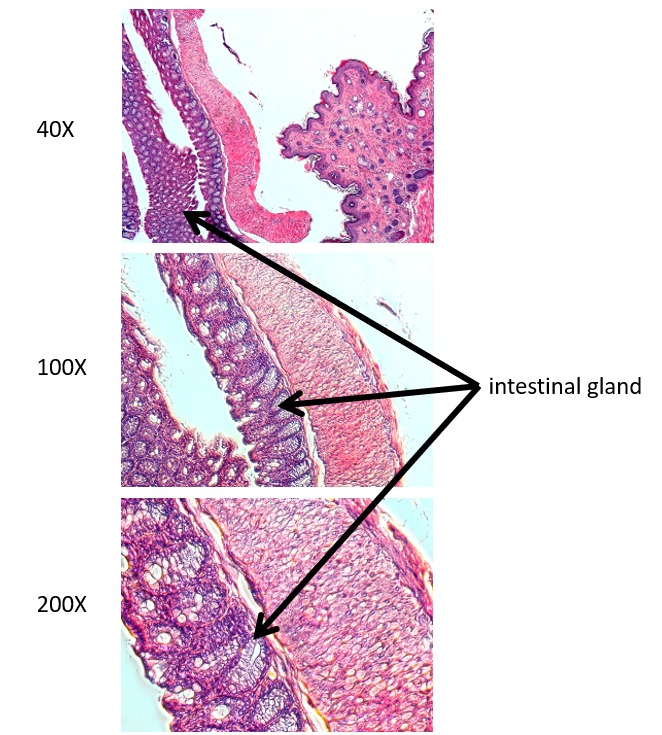


Supplementary Figure 2

Histopathologic examination of murine intestines from the control group. Intact intestinal gland and submucosal structure are presented. There were no signs of adenocarcinoma.

**Supplementary Figure 3**


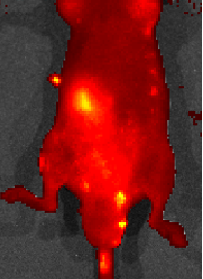


Supplementary Figure 3

Twenty-four hours after oral administration of the liposome solution with DiD fluorescent dye, fluorescence could be detected. The result suggested that the liposome structure was stable in the murine intestinal tract.

**Supplementary Figure 4**


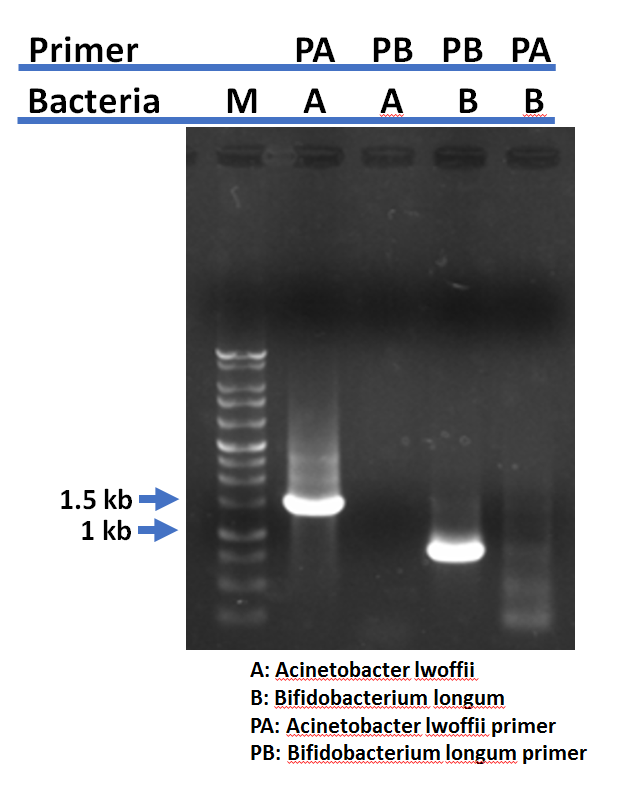


Supplementary Figure 4

The positive control exhibited a standard image after DNA analysis. The *Acinetobacter lwoffii* DNA marker appeared at 1500 bp, and *Bifidobacterium longum* appeared at 831 bp.

**Supplementary Tables**

**Table S1 Summary of the primers of the two target bacteria**

|  | *A. Iwoffii* | *B. longum* |
| --- | --- | --- |
| Bacteria mark | A | B |
| Primer mark | PA (primer for *A.* *lwoffii*) | PB (primer for *B. longum*) |
| PCR product size | 1500 bp | 831 bp |

PCR, polymerase chain reaction.
